# Holi colours contain PM10 and can induce pro-inflammatory responses

**DOI:** 10.1186/s12995-016-0130-9

**Published:** 2016-09-10

**Authors:** Katrin Bossmann, Sabine Bach, Conny Höflich, Kerttu Valtanen, Rita Heinze, Anett Neumann, Wolfgang Straff, Katrin Süring

**Affiliations:** 1 Environmental Medicine and Health Effects Assessment, Federal Environment Agency, Corrensplatz 1, 14195 Berlin, Germany; 2 Microbiological Risk Assessment, Federal Environment Agency, Corrensplatz 1, 14195 Berlin, Germany; 3 Toxicology of drinking water, Federal Environment Agency, Heinrich-Heine-Str. 12, 08645 Bad Elster, Germany

**Keywords:** Holi colours, PM10, Pro-inflammatory response, TNF-α, IL-6, IL-1β, Oxidative burst

## Abstract

**Background:**

At Holi festivals, originally celebrated in India but more recently all over the world, people throw coloured powder (Holi powder, Holi colour, Gulal powder) at each other. Adverse health effects, i.e. skin and ocular irritations as well as respiratory problems may be the consequences. The aim of this study was to uncover some of the underlying mechanisms.

**Methods:**

We analysed four different Holi colours regarding particle size using an Electric field cell counting system. In addition, we incubated native human cells with different Holi colours and determined their potential to induce a pro-inflammatory response by quantifying the resulting cytokine production by means of ELISA (Enzyme Linked Immunosorbent Assay) and the resulting leukocyte oxidative burst by flow cytometric analysis. Moreover, we performed the XTT (2,3-Bis-(2-methoxy-4-nitro-5-sulfophenyl)-2H-tetrazolium-5-carboxanilide) and Propidium iodide cytotoxicity tests and we measured the endotoxin content of the Holi colour samples by means of the Limulus Amebocyte Lysate test (LAL test).

**Results:**

We show here that all tested Holi colours consist to more than 40 % of particles with an aerodynamic diameter smaller than 10 μm, so called PM10 particles (PM, particulate matter). Two of the analysed Holi powders contained even more than 75 % of PM10 particles.

Furthermore we demonstrate in cell culture experiments that Holi colours can induce the production of the pro-inflammatory cytokines TNF-α (Tumor necrosis factor-α), IL-6 (Interleukine-6) and IL-1β (Interleukine-1β). Three out of the four analysed colours induced a significantly higher cytokine response in human PBMCs (Peripheral Blood Mononuclear Cells) and whole blood than corn starch, which is often used as carrier substance for Holi colours. Moreover we show that corn starch and two Holi colours contain endotoxin and that certain Holi colours display concentration dependent cytotoxic effects in higher concentration. Furthermore we reveal that in principle Holi colours and corn starch are able to generate an oxidative burst in human granulocytes and monocytes. In Holi colour 1 we detected a fungal contamination.

**Conclusions:**

Some of the observed unwanted health effects of Holi colours might be explained by the high content of PM10 particles in conjunction with the possible induction of a pro-inflammatory response and an oxidative leukocyte burst.

## Background

Holi is a traditional Hindu festival originated in India. Lasting for several days in spring and celebrating the victory of good over evil it is a very colourful event. Differences in people’s caste, age, sex and status seem to be abrogated by throwing Gulal, a special coloured powder, at each other to make everybody and everything look equal. About 3 years ago, the traditional Indian festival was transferred to Europe and adapted to a commercial party event. Meanwhile it takes place in many different cities all over the world: people listen to music, dance and throw coloured powder. This so called Holi powder/Holi colour/Gulal powder or Micro Confetti is distributed by different companies. The quality and the amount of information given on the outer packaging about the ingredients of the particular Holi colour are mostly very poor. So far, there is no European or US regulation about Holi colours and it is not clear whether they should be categorised for instance as cosmetic products or general consumer products. Although many manufacturers claim that their products are harmless to health and environment, organisers of Holi festivals often recommend wearing eye and respiratory protection (like goggles and face masks) and advise people with respiratory disorders not to participate. There are several reports of adverse health effects probably caused by the use of Holi powder: various forms of cutaneous diseases [1] as well as ocular irritations [1, 2] and a case of periorbital necrotizing fasciitis [3] have been described after exposure to Holi colours. In addition, high total suspended particulate concentrations occurred during Holi events [4] that may also cause or aggravate adverse health effects like respiratory irritations.

To assess possible hazards to human health, we ordered four different Holi powder products of three different distributors via the internet and analysed them regarding particle size and their potential to induce pro-inflammatory responses in cell culture systems. Cell-based in vitro assays have been used widely to imitate in vivo situations [5, 6]. For example the whole blood assay was used to describe pro-inflammatory activity of dust sample extracts [7]. Moreover, we tested the cytotoxic potential of corn starch and the different Holi colours, analysed the endotoxin and mould content of corn starch and the Holi colours and screened for corn starch-/Holi colour-leukocyte interactions in vitro.

## Methods

### Holi colours

Four different Holi colour products were ordered via a renowned internet retailer. General information like producing country, ingredients or indications relevant for impacts on health either given on the internet or on the outer packaging of the product are shown in Table 1. As corn starch is often used as carrier substance for Holi colours, commercially available corn starch (RUF Lebensmittelwerk KG, Quakenbrück, Germany) designated as food ingredient was purchased to serve as a control.

**Table 1:** Available information on the different Holi colours, either communicated with the product itself or online

Holi Colour Number	1	2	3	4
Shade/ characterisation of colour	black/ Holi colour, perfumed natural colour, Holi powder Gulal	pink/ Holi powder (Holi gulal Holi colour), used at Holi Festivals	orange/ “Effect colour powder”, optically like Holi powder/ Holi colours	green/ Holi colour, Gulal festival
Produced in	India	Germany	n.s.	n.s.
MSDS available	n.s.	yes	n.s.	n.s.
EU Regulation	n.s.	according to Art. 19 EU regulation of cosmetic products	n.s.	n.s.
Ingredients	natural colour, plant based	corn starch, water, hydrated silica, sodium chloride, sodium sulphate, E127	n.s.	flour powder, dyed
Recommended application	Holi open air festivals, creative areas	Holi festivals, for throwing up into the air, sprinkle powder onto hands and throw up into air (away from body) for adult use only	for decor use or throwing up into the air	for decor use or throwing up into the air
Warning	environmental friendly, mild on skin, nontoxic	don’t use with a history of asthma and allergies, avoid contact with mucus membranes, wear mouth, nose and eye protection, not edible	don’t use at festivals or on skin, not classified as cosmetic product	don’t use at festivals or on skin, not classified as cosmetic product

*n.s.* not specified, *MSDS* material safety data sheet, *number 2 and 3* same distributor

### Electric field cell counting system (CASY®) of corn starch and Holi particles

We used a CASY® cell counter (Schärfe System GmbH, CASY Cell Counter + Analyser System, Model TTC) with a 60 μm measuring capillary to determine particle size and number of (i) BD Cytometer Setup and Tracking beads of 2 and 3 μm diameter (Becton Dickinson, Lot: 22680), (ii) blank calibration particles (BD Biosciences, Lot: 63100) of 6.0–6.4 μm diameter, (iii) corn starch and (iiii) four different Holi colours. Respective particles were suspended in PBS (Phosphate buffered saline) (Biochrom, Berlin, Germany) and diluted with CASY® ton (La Roche Diagnostics GmbH, Basel, Switzerland) according to the manufacturer’s instructions for measuring. Particle count was evaluated in a size range between 0.7 and 30 μm.

### Stimulation of PBMCs

Blood was withdrawn under authorised supervision from six healthy donors who had given their informed consent. PBMCs were isolated from lithium heparin anti-coagulated blood by Ficoll-Paque™ Plus (GE Healthcare Bio-Sciences AB, Uppsala, Sweden) density gradient centrifugation and resuspended in VLE RPMI 1640 (Very Low Endotoxin) media (Biochrom AG, Berlin, Germany) supplemented with 10 % (v/v) FCS (Fetal Calf Serum), 2 mM L-glutamine (Sigma Aldrich, Chemie GmbH, München, Germany) and 1 % (v/v) penicillin/streptomycin (Biochrom AG, Berlin, Germany). 500 μl of a suspension of PBMCs (2 × 10^6^ cells/ml) were incubated in sterile 24-well plates (Multiwell™, Falcon®, Becton Dickinson Labware, NJ, USA) for 4 h at 37 °C and 5 % CO_2_ with corn starch and the four different Holi colours (see above), respectively, each at 1.5 × 10^6^ particles/ml. The utilised concentration of the Holi colours and corn starch arose from dose-effect experiments that we performed with ambient dust (data not shown). Cells treated with PBS only (7.5 % v/v) served as negative control. Cells treated with LPS (Lipopolysaccharide) (Enzo Life Sciences, NY, USA) at 100 ng/ml served as positive control. Samples were centrifuged for five minutes at 300 g and supernatants were transferred to fresh cell culture plates and frozen at −20 °C until further analysed.

### Stimulation of whole blood

Blood was withdrawn under authorised supervision from six healthy donors who had given their informed consent. 100 μl of a PBS suspension containing corn starch, the four different Holi colours or LPS respectively was mixed with 1 ml of a 0.9 % NaCl solution (Fresenius Kabi Deutschland GmbH, Bad Homburg, Germany). Subsequently 100 μl of lithium heparin anti-coagulated whole blood was added, samples were mixed carefully and stimulated in sterile 1.5 ml safe-lock tubes (Biopur, Eppendorf AG, Hamburg, Germany) overnight at 37 °C and 5 % CO_2_. The final concentration of corn starch and the four different Holi colours was 6.8 × 10^5^ particles/ml and for LPS it was 45.45 ng/ml. PBS only served as negative control. Samples were then mixed carefully and centrifuged for 5 min at 300 g and supernatants were transferred to fresh Eppendorf tubes and frozen at −20 °C until further analysed.

### Analysis of cytokine production by ELISA

The concentration of the cytokines TNF-α, IL-6 and IL-1β in the supernatant was determined using commercially available ELISA kits (R&D Systems, Minneapolis, USA) following the manufacturer’s instructions. PBMC samples were blocked with 300 μl 1 % BSA (Bovine Serum Albumin) (Sigma Aldrich, St. Louis, USA) in PBS and whole blood with 300 μl 3 % BSA in PBS.

ELISA data are presented as mean ± SD (standard deviation) values from six independent experiments. Statistical analysis was performed using SPSS software version 18 (SPSS Inc., Chicago, USA). The Wilcoxon matched pairs signed-ranked test was used to test for group differences. *P*-values < 0.05 (two-tailed) were considered as significant.

### Analysis of endotoxin content of samples by Limulus Amebocyte Lysate test (LAL test)

The endotoxin content of an unstimulated control, Holi colours 1–4, corn starch and LPS was determined using a commercially available Limulus Amebocyte Lysate test kit (Pierce® LAL Chromogenic Endotoxin Quantitation Kit, Pierce Biotechnology, Rockford, USA) as described by the manufacturer. Corn starch and Holi colour samples were applied in duplicate at 1.5 × 10^6^ particles/ml, LPS at 100 ng/ml.

### XTT test

100 μl HepG2 cells (c = 2.5 × 10^5^ cells/ml, Leibniz Institut, DSMZ, Braunschweig, Germany) were disseminated in RPMI 1640 media (Sigma-Aldrich Chemie GmbH, München, Germany) supplemented with 10 % (v/v) FCS (Sigma-Aldrich Chemie GmbH, München, Germany) in a 96 well plate (Cellstar, Greiner Bio One International GmbH, Leipzig, Germany) and incubated over night at 37 °C and 5 % CO_2_ for adherence. Then media was discarded and replaced by 100 μl RPMI 1640 supplemented with 1 % (v/v) FCS. Subsequently 100 μl of a single Holi colour or corn starch suspension in PBS were added at a final concentration of 1.7 × 10^6^, 2.5 × 10^6^, 5 × 10^6^ and 1 × 10^7^ particles/ml, each in quadruplicate. After 4 h of incubation cells were washed with PBS and supplemented with 100 μl of fresh RPMI 1640 containing 1 % (v/v) FCS. Then 50 μl of XTT reagent was added according to the manufacturer’s instructions (Cell Proliferation Kit II (XTT), product number 11465015001, Sigma-Aldrich Chemie GmbH, München, Germany). After 3 h of incubation at 37 °C and 5 % CO_2_ samples were mixed thoroughly and measured on a microplate reader (Anthos Zenyth 200RT, Biochrom, Cambridge, UK) at 490 nm (reference wavelength 690 nm). Vitality of cells was calculated in percent in relation to an unstimulated control sample.

### Propidium iodide test

500 μl Jurkat cells (c = 1 × 10^6^ cells/ml, Leibniz Institut, DSMZ, Braunschweig, Germany) in RPMI 1640 media supplemented with 10 % (v/v) FCS were disseminated in a 24 well plate (Cellstar, Greiner Bio One International GmbH, Leipzig, Germany). Then 500 μl of single Holi colour or corn starch suspension in PBS were added to reach a final concentration of 1.7 × 10^6^, 2.5 × 10^6^, 5 × 10^6^ and 1 × 10^7^ particles/ml, respectively. Samples were incubated for 4 h and for 20 h at 37 °C and 5 % CO_2_. After washing with 1 ml PBS cells were suspended in 1 ml fresh media without FCS and phenol red (RPMI 1640, Sigma-Aldrich Chemie GmbH, München, Germany), incubated for 15 min with 8 μg/ml propidium iodide (Sigma-Aldrich Chemie GmbH, München, Germany) in the dark at room temperature and analysed by Flow Cytometry (FACS Calibur, BD Biosciences). Vitality of cells was calculated in percent in relation to an unstimulated control sample.

### Light Microscopy of corn starch and Holi colours together with human leukocytes

Blood was withdrawn under authorised supervision from two healthy donors who had given their informed consent. Cells were treated as described for the stimulation of PBMCs and stimulation of whole blood. After the 4 h, respectively overnight incubation, 20 μl of the cell-corn starch or cell-Holi colour mixture was pipetted onto an object slide, covered with a cover slip and immediately analysed under a light microscope (Axio Vert A1 with Axiovision Rel.4.8.2 software, Zeiss, Jena, Germany). Some of the whole blood samples were also stained with Syto 9 (Life Technologies, USA,) for 10 min as described by the manufacturer and analysed under the microscope in the green fluorescence channel.

### Phagoburst Assay

Blood was withdrawn under authorised supervision from four healthy donors who had given their informed consent. Phagoburst™ reagent Kit (Glycotope Biotechnology, Germany) was used to measure the oxidative burst activity of monocytes and granulocytes in human whole blood as described by the manufacturer except for the incubation time at 37 °C which was reduced to overall 12 min instead of 20 min due to a reduced cell viability observed at the longer incubation time. Additionally to the reagents in the kit, corn starch and Holi colour 1 were incubated with the heparinised human whole blood. The concentrations of corn starch and Holi colour 1 corresponded to the E.coli (Escherichia coli) concentration of 2 × 10^9^ particles/ml. Samples remaining on ice served as negative control.

### Analysis of mould content

100 μl of each Holi colour and corn starch suspension (c = 1.5 × 10^6^ particles/ml) was inoculated under sterile conditions on malt extract agar (MEA, OXOID GmbH, Wesel, Germany) and Dichloran 18 % Glycerol agar (DG18, heipha Dr. Müller GmbH, Eppelheim, Germany). The agar plates were incubated at 25 °C and 36 °C for 10 days, respectively. The micro-morphology of selected colonies was analysed by optical microscopy (Axioskop 40 FL, Carl Zeiss MicroImaging GmbH, Göttingen, Germany) at 200× and 1000× magnification and subsequently photographed with a digital camera (Canon PowerShot Gs, Canon Deutschland, Krefeld, Germany).

## Results

### The tested Holi colours consist of more than 40 % PM10 particles

**Fig. 1:**
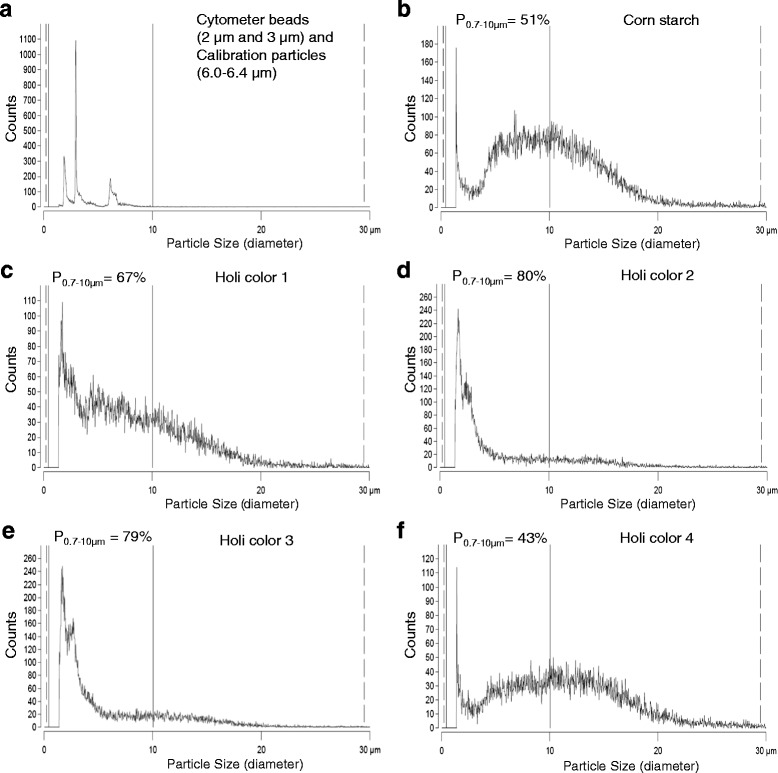
Particle size distribution of beads, corn starch and Holi colours. Illustrated is the particle size distribution of commercially available beads of 2 µm, 3 µm and 6-6.4 µm (**a**), corn starch (**b**) and 4 different Holi colours (**c-f**). The gate _P0.7-10µm_ indicates the percentage of particles bigger than the detection limit of 0.7 µm but smaller than 10 µm in diameter

**Fig. 2:**
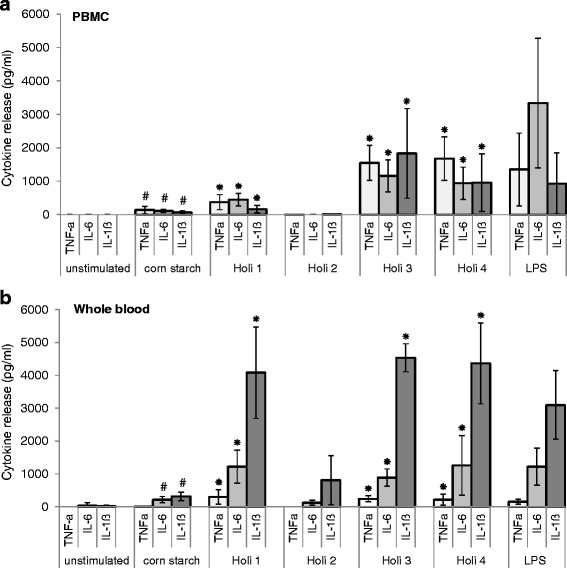
Induction of pro-inflammatory cytokines by corn starch and Holi colours. Shown is the production of TNF-α, IL-6 and IL-1β induced by corn starch and Holi colours 1-4 in (**a**) PBMCs and (**b**) whole blood. PBS served as negative and LPS as positive control. Each bar represents the mean value of six independent experiments representing 6 different probands. The standard deviation is depicted by the error bars. # significant (*p* < 0.05) when compared to unstimulated cells. * significant (*p* < 0.05) when compared to unstimulated cells and cells treated with corn starch

We analysed commercially available particles of known size, corn starch - often used as Holi colour carrier substance - and four different Holi powders regarding particle size and number by use of a CASY® Cell Counter (Fig. 1). BD Cytometer Setup and Tracking beads of 2 and 3 μm diameters mixed with blank calibration particles of 6.0–6.4 μm diameter show precise peaks at the respective sizes (Fig. 2a), thus confirming CASY® Technology as an accurate method regarding particle size characterisation. To identify the inhalable fraction of the analysed particles the percentage of counts <10 μm in diameter was determined. Because of background artefacts the range from 0 to 0.7 μm was excluded. This resulted in a gate set between 0.7 and 10 μm (P_0.7–10μm_ in Fig. 1). The content of particles between 0.7 and 10 μm in diameter varied between the analysed powders: corn starch consisted to 51 % of particles >0.7 μm and <10 μm (Fig. 1b) while the content of particles between 0.7 and 10 μm in the Holi colours ranged from 43 to 80 % (Fig. 1c–f). Thus all four Holi powders consisted of more than 40 % potentially inhalable particles that might reach the lower respiratory tract.

### Holi colours can induce the production of TNF-α, IL-6 and IL-1β

To assess the pro-inflammatory capacity of the different Holi colours, we tested their potential to induce the production of the pro-inflammatory cytokines TNF-α, IL-6 and IL-1β. We used two different cell culture systems and two different incubation times: human PBMCs were incubated for 4 h and human whole blood was incubated overnight with corn starch and four different Holi colours. The resulting cytokine production was analysed by ELISA. Cells treated with PBS only served as unstimulated control and cells treated with LPS served as positive control.

In both cell culture systems, cells incubated with corn starch or Holi colours showed a higher production of TNF-α, IL-6 and IL-1β than unstimulated cells (Fig. 2). This increase was significant (*p* < 0.05) for all analysed cytokines and all Holi samples except for Holi colour 2. Holi colours 1, 3 and 4 induced a significantly higher cytokine production than corn starch (*p* < 0.05 for TNF-α, IL-6 and IL-1β). Corn starch also slightly increased the production of TNF-α, IL-6 and IL-1β when compared to the cells treated with PBS only (Fig. 2). This increase was significant (*p* < 0.05) for TNF-α, IL-6 and IL-1β in PBMCs and for IL-6 and IL-1β in the whole blood experiments.

### Corn starch and Holi colours 3 and 4 contain endotoxin

**Fig. 3:**
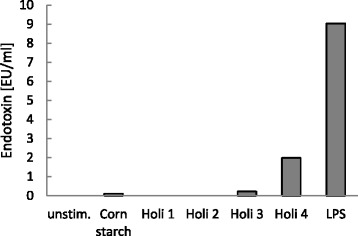
Endotoxin content of corn starch and Holi colours 1–4. The endotoxin content of corn starch and Holi colours 1–4 was determined by LAL test. The resulting endotoxin amount was measured in Endotoxin Units per ml (EU/ml). Samples were applied in the same concentration as used for stimulation of PBMCs (corn starch and Holi colours 1–4: c = 1.5 × 10^6^ particles/ml, LPS: 100 ng/ml)

As endotoxin is a potent inducer of pro-inflammatory cytokines [8], an endotoxin quantification test was performed to determine the endotoxin content of corn starch and the different Holi colours. Hereby, the same particle and LPS concentration was used as for the stimulation of PBMCs. In the unstimulated control and in Holi colours 1 and 2 no endotoxin was detectable, while corn starch exhibited an endotoxin level of 0.11 EU/ml (Endotoxin Units/ml), Holi colour 3 of 0.23 EU/ml, Holi colour 4 of 1.99 EU/ml and the positive control LPS of 9 EU/ml (Fig. 3).

### Holi colour 2 and 3 show cytotoxicity with increasing particle concentration

To determine possible cytotoxic effects of Holi colours and corn starch we performed two different cytotoxicity tests: the XTT test and the Propidium iodide test. The XTT test revealed that neither corn starch nor Holi colours 1, 3 or 4 exhibited cytotoxicity towards HepG2 cells in the concentration range tested. Holi colour 2 showed no cytotoxicity in the lowest concentration (1.7 × 10^6^ particles/ml) but cytotoxicity increased with raising particle concentration (data not shown). In the Propidium iodide test corn starch and Holi colours 1, 2 and 4 did not show any cytotoxic effects. Holi colour 3 displayed increasing cytotoxicity with increasing particle concentration (data not shown). But due to interferences with the fluorescence of the Holi colours and the fluorescence to be measured by flow cytometry, the evaluation of the Propidium iodide test was very difficult and the data should be considered preliminary.

### Holi colour particles are associated with human leukocytes in the microscopic image

Human PBMCs and human whole blood were incubated for 4 h and overnight with corn starch and four different Holi colours to determine the fate of the particles when administered to cells and to test for possible cell-particle-interactions.

**Fig. 4:**
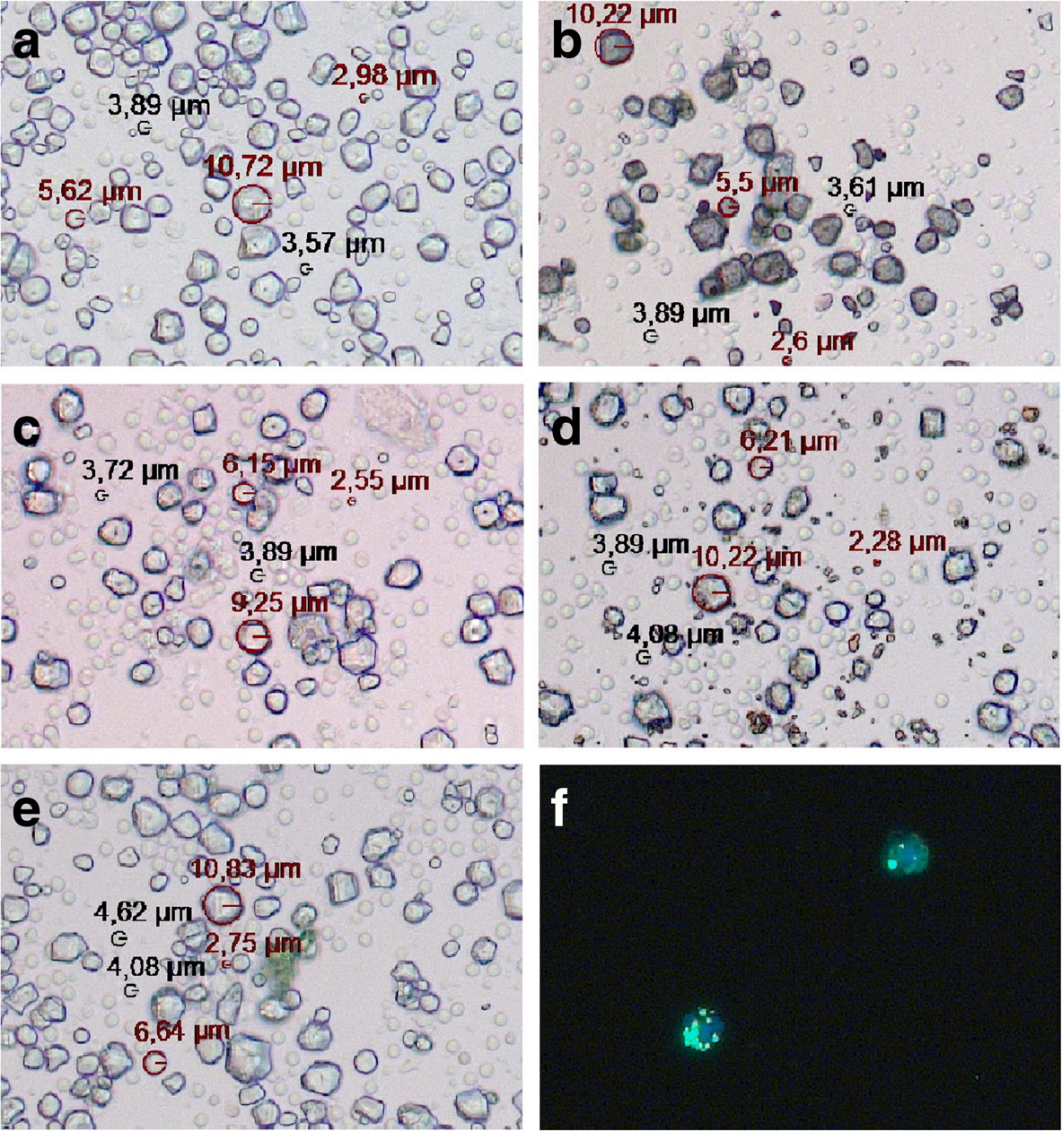
Light Microscopic images of human PBMCs together with Holi colours or corn starch. **a**–**e** shows 200 fold magnifications of native preparations of human PBMCs incubated for 4 h together with corn starch (**a**), Holi colour 1 (**b**), Holi colour 2 (**c**), Holi colour 3 (**d**) and Holi colour 4 (**e**). Cell sizes are indicated in black and particle sizes in red and the respective radiuses are stated. (Note that very small particles could not be indexed.) **f** displays a 400 fold magnification of cells incubated overnight with Holi colour 3, then stained with the nucleic acid dye Syto 9 and analysed in the green fluorescence channel

Light Microscopy of human PBMCs together with Holi colours and corn starch demonstrated the irregular shape of the bigger particles (radius of about 3 to 10 μm) and showed an even distribution of cells and particles (Fig. 4a–e). Due to the autofluorescence of Holi colours 2, 3 and 4, Holi particles could be visualised by Fluorescence Microscopy. When cells were stained with the nucleic acid dye Syto 9, this revealed a close particle-cell-association of the smaller Holi particles with human leukocytes as shown in Fig. 4f for Holi colour 3.

### Holi colour 1 and corn starch can induce an oxidative burst in human granulocytes and monocytes

**Fig. 5:**
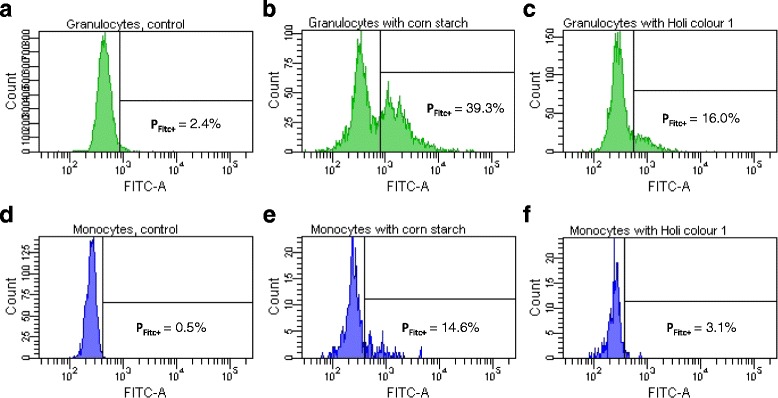
Induction of leukocyte oxidative burst by corn starch and Holi colour1. Exemplified is the illustration of the percentage of granulocytes (**a-c**) and monocytes (**d-f**) which produce reactive oxidants in one proband after **a** and **d**: incubation with corn starch at 0 °C (negative control); **b** and **e**: incubation with corn starch at 37 °C; **c** and **f**: incubation with Holi colour 1 at 37 °C. The gate P_FITC+_ displays the particles considered FITC positive and respective percentages are given

We incubated human whole blood (*n* = 4) with corn starch and the different Holi colours and determined the resulting leukocyte oxidative burst by flow cytometric analysis. Due to interference of the autofluorescence of Holi colours 2, 3 and 4 with the fluorescence measured as a result of the oxidative burst, only corn starch and Holi colour 1 could be tested. Preliminary data revealed that 29.5–39.3 % of human granulocytes and 11.4–25.7 % of human monocytes produced reactive oxidants after incubation with corn starch. Holi colour 1 induced an oxidative burst in 16–24.1 % of human granulocytes and 2.7–4.3 % of human monocytes (Fig. 5).

### Holi colour 1 contains mould fungi

**Fig. 6:**
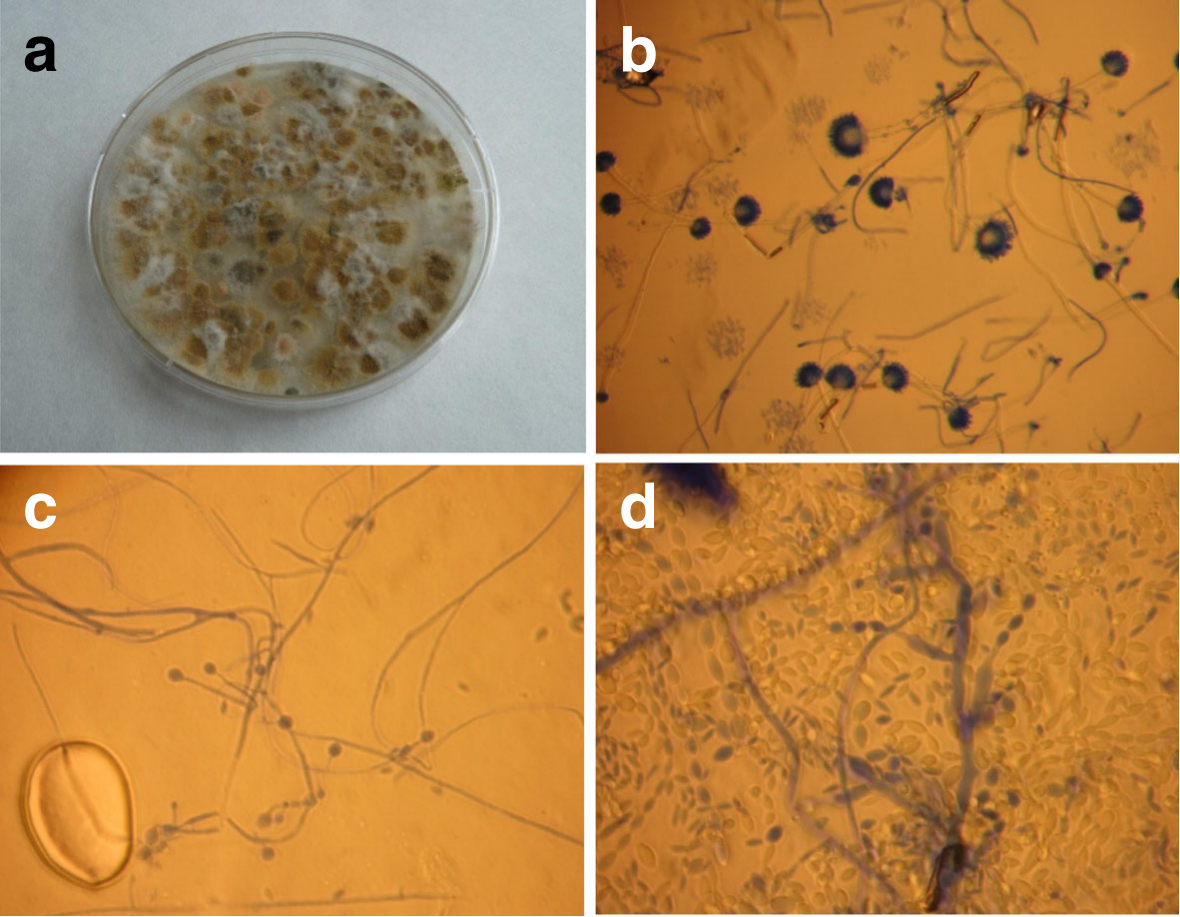
Holi colour 1 contains mould. **a** exhibits a photograph of a DG18 agar plate after inoculation with Holi colour 1 and incubation for 10 days at 25 °C. **b**–**d** displays microscopic images of different isolates of mould fungi (**b** *Aspergillus sp.*, **c** unidentified *Zygomycete* species, **d** unidentified *Paecilomyces/Bossychlamus* species) found in Holi colour 1 (Magnification: 200×)

Agar plates inoculated with Holi colour 1 exhibited fungal growth of several macromorphologically different colonies (Fig. 6a). Fungal growth was not observed on plates inoculated with Holi colours 2–4 and corn starch (data not shown).

By micromorphological analysis different isolates of *Aspergillus spp*., unidentified *Zygomycete* species and unidentified *Paecilomyces/Bossychlamus* species were detected (Fig. 6b–d).

## Discussion

Holi colours can potentially be harmful to human health: they contain a considerable amount of particles with an aerodynamic diameter smaller than 10 μm and at least in vitro they show a close association with human leukocytes, a pro-inflammatory potential, they can have cytotoxic effects in higher concentration and can induce an oxidative burst in human granulocytes and monocytes.

Extensive use of Holi powder at Holi festivals will result in a considerable increase in PM10 concentrations in ambient air. Adverse health effects due to long-term exposure to high PM10 concentrations are widely known: increasing concentrations of particulate matter are related to a higher cardiovascular and respiratory morbidity and mortality [9–13]. Also, there is evidence for short-term effects of air pollutants: increased PM10 concentrations were also associated with an increase in daily mortality [14, 15]. In addition, a positive association of dust storms with mild asthma manifestations in children, as indicated by medication purchases, could be shown recently [16].

In this study the particle size of the examined Holi colours differed (Fig. 1). While Holi colours 2 and 3 consisted of about 80 % of particles smaller than 10 μm in diameter - with the majority of particles being even smaller than 5 μm (Fig. 1d–e) -, the particle size of Holi colours 1 (Fig. 1c) and 4 (Fig. 1f) ranged between 0.7 and 20 μm with a PM10 content of 67 and 43 %, respectively.

The size resolution in the CASY® analysis confirmed approximately the average particle size of corn starch (Fig. 1b) stated in the literature: 15 μm in diameter [17]. But except for Holi colour 4 the other colours contained a greater number of smaller particles. Thus, the corn starch used for Holi colour production might be somehow modified during the colour production process or mixed with other substances. For the Holi colours 1, 3 and 4 it is also possible that they comprise another carrier substance, e.g. rice flour or other anticaking agents.

It is difficult to compare the specific exposure at/or in the vicinity of Holi festivals with the “normal” ambient PM10 exposure i.e. caused by road traffic or combustion processes. The composition of the particulate matter seems to have a great influence on the biological effects as i.e. assessed by cytokine induction [18]. Also epidemiological data show that not only the particle size but furthermore the chemical composition accounts for harmful health effects [19, 20].

In this study, the four different Holi colours induced diverging amounts of pro-inflammatory cytokines as shown in the cell culture experiments (Fig. 2). Differences in the proportion of particle size alone cannot account for the different levels of pro-inflammatory potential: although Holi colours 2 and 3 had nearly the same amount of particles >0.7 μm and <10 μm (about 80 %), they induced significantly diverging amounts of TNF-α, IL-6 and IL-1β.

In general as compared to PBMCs, which were stimulated for 4 h, (Fig. 2a) we found a decrease in TNF-α production and an increase in IL-1β production in the whole blood experiments (Fig. 2b), where cells were incubated with the respective substances overnight. This corresponds well with the kinetics of release of TNF-α and IL-1β in whole blood and PBMCs: TNF-α reaches maximal levels within 6 h of stimulation, whereas IL-1β reaches maximal levels between 12 and 16 h [21, 22].

The release of the pro-inflammatory cytokines TNF-α, IL-6 and IL-1β by human cells stimulated in vitro with particulate matter PM10 or nanoparticles has been shown in various studies [18, 23–27]. The fact that exposure to swine dust in vivo also leads to an increase of TNF-α, IL-6 and IL-1β in peripheral blood of healthy volunteers [28] suggests, that these cytokines might also be able to mediate this kind of inflammatory response in vivo.

Endotoxin is a potent inducer of various pro-inflammatory cytokines [8]. To elucidate the role of endotoxin in the cytokine induction of the Holi colours, we measured the amount of endotoxin in the different Holi samples by means of the LAL test. We did not detect any endotoxin in Holi colours 1 and 2, but discovered endotoxin in corn starch as well as in Holi colours 3 and 4, albeit these amounts of endotoxin are much below the endotoxin level measured in LPS (Fig. 3). But since already very low amounts of endotoxin in the picogramme range (which represent Endotoxin units of 1 EU/ml or even below according to the manufacturer’s product guide of the LAL test used here) can be responsible for considerable cytokine secretions the observed cytokine induction of Holi colours 3 and 4 might be caused by endotoxin. (We performed dose response tests for endotoxin levels and cytokines of interest. The results will be subject of a separate publication.) Nevertheless as Holi colour 1 contains no endotoxin and Holi colours 3 and 4 contained lesser endotoxin than LPS but induced a stronger cytokine release, we argue that also other factors than endotoxin might drive the cytokine answer.

Hansen et al. have shown that the potency of endotoxin from different Gram-negative bacteria in the LAL-assay is not closely correlated with the potency of the endotoxin to induce IL-8 (Interleukine-8) secretion from pulmonary epithelial cells [29]. This observation might be true for other pro-inflammatory cytokines as well. Hence the results of the LAL-test do not necessarily have to correlate with the cell culture data. This would also explain why corn starch, which contained endotoxin, only induces a very low cytokine release.

The XTT test and Propidium iodide test showed possible cytotoxic effects of Holi colours 2 and 3 with increasing Holi colour concentration, while corn starch and Holi colours 1 and 4 did not display any cytotoxicity thus underlying the heterogeneity of Holi colours. Since the observed cytotoxicity occurs only at higher concentrations or is rather less pronounced in the lowest concentration tested, respectively, their relevance to health regarding a real Holi festival situation would need further investigation.

Although fluorescence microscopy (Fig. 4f) showed a close particle-cell-association the nature of this association remains unclear: are the particles bound to the cell surface and if so through what kind of receptor or are the particles phagocytosed by the cell? Due to the impossibility of quenching the autofluorescent signal of the Holi colours in the flow cytometric analysis an exact discrimination of particles bound to the cell surface and particles phagocytosed by the cell was not feasible and we were not able to show an internalisation of Holi particles directly (data not shown). But as corn starch and to a lesser extent also Holi colour 1 induced an oxidative burst in human granulocytes and monocytes (Fig. 5), one can speculate that these cells might engulf the particles. However, the limitation remains, that the Phagoburst assay is an in vitro setup that is very different from the situation in vivo regarding i.e. time of exposure and particle concentration. Still, phagocytosis and processing of particulate matter by the macrophages in the lung is an established phenomenon which leads to diverse immunological responses and cytokine release [30].

*Aspergillus* spp. as well as other filamentous fungi could be detected in Holi colour 1 (Fig. 6). Moulds in general pose a risk to human health, can be involved in disorders of the respiratory tract and may contribute to the manifestation of asthma and allergies [31]. As Kawakami et al. [32] and Wykoff et al. [33] reported, *Aspergillus* and *Paecilomyces* species can play a role in ocular infections with soft contact lens use as one predisposing factor. In the context of numerous ocular irritations reported by medical services on Holi festivals (Becker et al., *submitted*) this detection of fungi may be of clinical relevance. Deeper insights in the microbiological contamination profile of Holi colours might be of interest for future investigations.

Not much is known about the ingredients of the Holi colours (see Table 1). Even the carrier substance often remains unknown. As the composition (carrier substance, colour pigments, anticaking agents, etc.) varies from colour to colour, also the observed in vitro effects differ. Similarly, underlying mechanisms as well as the generation of possible health effects might be diverse and dependent on the specific colour. However, these various modes of action might also work in an additive or synergistic fashion as i.e. endotoxin, fungal contamination and leukocyte oxidative bursts might potentially lead to the observed induction of pro-inflammatory cytokines. Further experiments are needed to elucidate the distinct underlying mechanisms in more detail.

## Conclusion

Holi festivals - with intense throwing of Holi powder in the air - are becoming popular all over the world. Although adverse health effects like skin, ocular or respiratory irritations may be the consequence, the hazard of Holi powder and possible underlying mechanisms of harmful effects have not been studied so far. We show here that the composition and the observed in vitro effects differ from colour to colour: Holi colours can contain up to 80 % of PM10 particles, they can have a pro-inflammatory and a cytotoxic potential, they can induce a leukocyte oxidative burst and they might be contaminated with mold fungi. These facts may account for some of the observed adverse health effects described by participants of Holi festivals and may improve the risk assessment of Holi colours.

## Abbreviations

BSA: Bovine Serum Albumin
E.coli: Escherichia coli
ELISA: Enzyme Linked Immunosorbent Assay
EU: Endotoxin Units
FCS: Fetal Calf Serum
IL-1β: Interleukine-1β
IL-6: Interleukine-6
IL-8: Interleukine-8
LAL: Limulus Amebocyte Lysate
LPS: Lipopolysaccharide
PBMCs: Peripheral Blood Mononuclear Cells
PBS: Phosphate buffered saline
PM: Particulate matter
SD: Standard deviation
TNF-α: Tumor necrosis factor α
VLE: Very low endotoxin
XTT: (2,3-Bis-(2-methoxy-4-nitro-5-sulfophenyl)-2H-tetrazolium-5-carboxanilide)
